# Genotypic and Phenotypic Characterization of *Stenotrophomonas maltophilia* Strains from a Pediatric Tertiary Care Hospital in Serbia

**DOI:** 10.1371/journal.pone.0165660

**Published:** 2016-10-31

**Authors:** Haowa Madi, Jovanka Lukić, Zorica Vasiljević, Marjan Biočanin, Milan Kojić, Branko Jovčić, Jelena Lozo

**Affiliations:** 1 University of Belgrade, Institute of Molecular Genetics and Genetic Engineering, Belgrade, Serbia; 2 Institute for Mother and Child Health Care of Serbia "Dr Vukan Čupić", Belgrade, Serbia; 3 University of Belgrade, Faculty of Biology, Belgrade, Serbia; Children's Hospital of Los Angeles, UNITED STATES

## Abstract

**Background:**

*Stenotrophomonas maltophilia* is an environmental bacterium and an opportunistic pathogen usually associated with healthcare-associated infections, which has recently been recognized as a globally multi-drug resistant organism. The aim of this study was genotyping and physiological characterization of *Stenotrophomonas maltophilia* isolated in a large, tertiary care pediatric hospital in Belgrade, Serbia, hosting the national reference cystic fibrosis (CF) center for pediatric and adult patients.

**Methods:**

We characterized 42 strains of cystic fibrosis (CF) and 46 strains of non-cystic fibrosis (non-CF) origin isolated from 2013 to 2015 in order to investigate their genetic relatedness and phenotypic traits. Genotyping was performed using sequencing of 16S rRNA gene, Pulse Field Gel Electrophoresis (PFGE) and Multi locus sequencing typing (MLST) analysis. Sensitivity to five relevant antimicrobial agents was determined, namely trimethoprim/sulfamethoxazole (TMP/SMX), chloramphenicol, ciprofloxacin, levofloxacin and tetracycline. Surface characteristics, motility, biofilm formation and adhesion to mucin were tested in all strains. Statistical approach was used to determine correlations between obtained results.

**Results:**

Most of the isolates were not genetically related. Six new sequence types were determined. Strains were uniformly sensitive to all tested antimicrobial agents. The majority of isolates (89.8%) were able to form biofilm with almost equal representation in both CF and non-CF strains. Swimming motility was observed in all strains, while none of them exhibited swarming motility. Among strains able to adhere to mucin, no differences between CF and non-CF isolates were observed.

**Conclusions:**

High genetic diversity among isolates implies the absence of clonal spread within the hospital. Positive correlation between motility, biofilm formation and adhesion to mucin was demonstrated. Biofilm formation and motility were more pronounced among non-CF than CF isolates.

## Introduction

*Stenotrophomonas maltophilia* is a ubiquitous environmental bacterium isolated from water, soil, animals and the plant rhizosphere and with ability to colonize moist surfaces in homes and hospitals [1]. During the last decade, it has been regarded as one of the emerging Gram-negative multi-drug resistant (MDR) organisms [2]. Being an opportunistic pathogen, it is commonly associated with healthcare-associated infections in debilitated and immunocompromised patients. However, community-acquired *S*. *maltophilia* infections have been occasionally reported [3]. The most common infections associated with *S*. *maltophilia* include respiratory tract infections, bacteremia, catheter-related infections and urinary tract infections [4]. In patients with cystic fibrosis (CF), *S*. *maltophilia* can colonize airways and cause chronic infections. However, the real contribution of the microorganism to CF pathogenesis still needs to be clarified [5, 6].

*S*. *maltophilia* exhibits high levels of intrinsic and acquired resistance to various antibiotics, considerably limiting treatment options [7–9]. This organism is intrinsically resistance to β-lactam antibiotics (including carbapenems), quinolones, aminoglycosides, and tetracyclines. Increased *S*. *maltophilia* isolation frequency in hospitals over the last decade might be attributed to the overuse and misuse of carbapenem antibiotics. Trimethoprim–sulfamethoxazole (TMP/SMX) has been recognized as the antibiotic of choice in the treatment of these bacteria [10]. However, recently reported increase in antimicrobial resistance of *S*. *maltophilia* notably to TMP/SMX is a matter of concern, so there is a compelling demand for new treatment strategies [2, 8].

Although *S*. *maltophilia* is not a highly virulent pathogen, its putative virulence factors, such as adhesion capacity, biofilm formation, hydrophobicity, motility and synthesis of extracellular enzymes, contribute to the inflammatory process [5]. *S*. *maltophilia* can adhere to different abiotic and biotic surfaces, and also adhere to and invade cultured epithelial respiratory cells [6]. Biofilms are highly organized multicellular communities of microorganisms. This multicellular existence appears to be preferred survival strategy of microbes, and is achieved through genetic components that regulate surface attachment, intracellular communications, and synthesis of extracellular polymeric substances. Biofim formation in *S*. *maltophilia* is affected by various factors, which still have not been well characterized. Nevertheless, recent studies have shown a correlation between mutations of *rmlA* and *rpfF* genes encoding glucose-1-phosphate thymidyl transferase and enoyl-CoA hydratase, respectively, and a decrease in biofilm formation [11, 12]. The *algC* gene responsible for the production of phosphoglucomutase (PGM) in *Pseudomonas aeruginosa* is homologous to *spgM* gene of *S*. *maltophilia* encoding PGM and involved in phosphomannomutase activities [13], which may have a role in biofilm formation.

The surfaces of gastrointestinal, respiratory and reproductive tracts are covered with mucus with barrier properties that is essential in preventing viruses and bacteria from entering the tissues. In certain lung diseases, such as CF, overproduction of abnormally thick and sticky mucus impedes the movement of cilia and prevents efficient elimination of trapped airborne bacteria. This allows for the accumulation of bacteria in the lower parts of the respiratory tract. Their ability to adhere to altered CF mucin leads to successful colonization of the respiratory tract, which is the first step in the pathogenesis of infections [14].

Although, *S*. *maltophilia* is an important nosocomial pathogen, little is known about the epidemiology of this organism in hospital settings in Serbia. Over the past few years, *S*. *maltophilia* has been recovered with increasing frequency at the Institute for Mother and Child Health Care of Serbia "Dr Vukan Čupić", a 400-bed University-affiliated pediatric tertiary care hospital in Belgrade, Serbia. This hospital is also the host for the national reference CF center providing care for pediatric and adult CF patients. The aim of the present study was to characterize 88 *S*. *maltophilia* clinical isolates of cystic fibrosis (CF) and non-cystic fibrosis origin (non-CF). In order to determine the microbiological characteristics of the isolates, we investigated their clonal relatedness and susceptibility to relevant antimicrobial agents, as well as the roles of cell surface properties, motility characteristics and selected genetic determinants in biofilm formation and adhesion to mucin. We also compared between the results obtained for CF and non-CF isolates.

## Materials and Methods

### Ethics Statement

The authors assert that all procedures contributing to this work comply with the ethical standards of the Ethics Committee of The Institute for Mother and Child Health Care of Serbia “Dr Vukan Čupić”on human experimentation and with the Helsinki Declaration of 1975, as revised in 2008. Since the analysis was performed retrospectively on isolates collected through routine clinical work and patient identifiable information was anonymized, no written or verbal informed consent to participate in this study from patient was necessary. The authors had no contact or interaction with the patients. Patient demographics anonymization was performed in two steps. First, personal data was coded by the head of the clinical microbiology laboratory (ZV) at the Institute for Mother and Child Health Care “Dr Vukan Čupić” where the isolates were obtained from, and secondly by assigning a different code by the principal investigator at the Institute of Molecular Genetics and Genetic Engineering (JL) where the molecular analysis was conducted. Ethics Committee of The Institute for Mother and Child Health Care of Serbia “Dr Vukan Čupić”specifically approved this study, approval No. 8/6a.

### Bacterial strains and species identification

*Stenotrophomonas maltophilia* isolates were collected over a 25-month period (from April 2013 to April 2015) during the course of routine health care at The Institute for Mother and Child Health Care of Serbia "Dr Vukan Čupić". At least one isolate per patient was included, as well as subsequent isolations that were coincidered phenotypically different or were recovered with a time interval of more than 6 months. Laboratory identification of the isolates was carried out using standard biochemical testing and automated Vitek 2 system (BioMérieux, Marcy l'Etoile, France). Before DNA extraction, the isolates were grown on Luria Bertani broth (LB) overnight at 37°C with aeration. Luria broth agar plates (LA) were prepared by adding 1.5% of agar to LB medium. All isolates were stored in LB with 15% glycerol at -80°C.

### Molecular identification of clinical isolates

The final molecular identification of the strain was performed by PCR for 16S rRNA gene with specific primers: UNI 16SF (5-GAG AGT TTG ATC CTG GC-3) and UNI 16SR (5-AGG AGG TGA TCC AGC CG-3). PCR products were purified with GeneJET PCR Purification Kit (Thermo Scientific, Lithuania) and sequenced by the Macrogen DNA sequencing service (Macrogen Inc., Netherlands). Obtained sequences were aligned in the NCBI database using BLAST. PFGE was performed as previously described [15]. Genomic DNA was digested with *Xba*I enzyme (Thermo Scientific, Lithuania), and obtained macrorestriction profiles were subject to statistical analysis. MLST was performed as was described in Kaiser et al [16] and the primers and protocols were downloaded from the website of the *S*. *maltophilia* MLST database (http://pubmlst.ors/smaltophilia/). Briefly, MLST was performed by PCR and sequencing of seven housekeeping genes: *atpD* (H (+)-transporting two-sector ATPase), *gapA* (NAD-dependent glyceraldehyde-3-phosphate dehydrogenase), *guaA* (GMP synthase), *mutM* (DNA-formamidopyrimidine glycosylase), *nuoD* (NADH dehydrogenase), *ppsA* (pyruvate, water dikinase), *recA* (RecA protein). Allele profiles obtained after sequencing were used to determine specific sequence type (ST) for analyzed isolates using MLST Database hosted by the University of Freiburg, Germany [16].

### Antimicrobial susceptibility testing

Antimicrobial susceptibility testing was performed according to the criteria of the Clinical and Laboratory Standard Institute (CLSI) [17]. Minimal inhibitory concentration (MIC) was determined by microdilution method for TMP/SMX (2, 4, 8, 16, 32 and 64 μg/ml). Disc diffusion method was used for susceptibility testing to chloramphenicol 30 μg, ciprofloxacin 5 μg, levofloxacin 5 μg and tetracycline 30 μg. For agents that specific CLSI breakpoints for *S*. *maltophilia* had not been published, the relevant criteria for *Pseudomonas aeruginosa* or *Escherichia coli* were used.

### Biofilm formation assay

Biofilm formation assay was performed as described by Stepanović et al [18]. Briefly, suspension in tryptic soy broth (TSB) of eash isolate was adjusted to the density of 0.5 McFarland (Biosan, Latvia). The cultures were then diluted 1: 100 in 200 μl TSB and were inoculated into the wells of a flat-bottomed polystyrene 96-well plate (Sarstedt, Newton, USA). *Pseudomonas aeruginosa* PAO1 was used as positive control and the negative control was sterile TSB media. Microtitre plates were incubated at 37°C for 24h, and wells subsequently washed three times with sterile PBS (pH 7.2). Adherent biofilms were fixed for 30 min at 65°C, stained for 30 min at room temperature with 200 μl of 0.1% crystal violet then rinsed in still water and dried at 65°C. Biofilms were resolubilized with 200 μl of solution containing 96% ethanol and acetone in ratio 4:1 for 15 min and the OD was read at 595 nm. The low cut-off (ODc) was calculated as the three standard deviations above the mean OD of control wells. Classification of strains was performed according to the following criteria: no biofilm producer (OD ≤ ODc), weak biofilm producer (ODc ˂ OD ≤ 2 x ODc), moderate biofilm producer (2 x ODc ˂ OD ≤ 4 x ODc) and strong biofilm producer (4 x ODc ˂ OD).

In addition, presence of *rmlA*, *spgM* and *rpfF* genes was determined by PCR with specific primers described previously by Pompilio et al. [19]. PCR products were sequenced by the Macrogen DNA sequencing service (Macrogen Inc., Netherlands). Obtained sequences were aligned in the NCBI database using BLAST.

### Surface characteristics and motillity assay

Hydrophobicity was determined as described by Begovic et al. [20]. Microbial adhesion to hexadecane (MATH) was analyzed for all 88 strains. The optical density of the initial (OD_0_) and extracted solution (OD_1_) was measured at OD_600nm_ (Ultrospec 3300 Pro, Amersham Biosciences). The fraction of bacteria adhering to hexadecane/water interface was calculated according to the following equation: θ = OD_0_ -OD_1_/OD_0_. To determine strain hydrophobicity previously defined values were used as reference values:0–35% marked low hydrophobicity, 36–70% medium hydrophobicity and 71–100% high hydrophobicity. Swimming and swarming motillity assays were performed as described by Pompilio et al. [19].

### Mucin adhesion assay

The ability of *S*. *maltophilia* strains to adhere to mucin was tested as described by Muñoz-Provencio et al. [21] with modifications. Flat-bottomed polystyrene 96-well plates (Sarstedt, Newton, USA) were covered with mucin (porcine stomach, Sigma, Germany) in 50 mM carbonate buffer pH 9.6 (mucin) at a concentration of 30 mg/ml, while the wells of control plates were filled with the same volume of 50 mM carbonate buffer (200 μl). Plates were incubated overnight at 4°C. After immobilization, wells were washed three times with PBS and blocked for 1 h with PBS plus 1% Tween 20. After washing, 200 μl aliquots of bacterial suspension adjusted to the turbidity of a 0.5 McFarland were added and plates were incubated for 2h at 37°C. Non-adherent cells were removed by washing three times with PBS plus 0.05% Tween 20 and the plates were dried at 65°C. Adhered cells were stained with 0.1 mg/ml of crystal violet (100μl/well) for 45 min. After six washes with PBS, the colorant was liberated with 50 mM citrate buffer pH 4.0 (100 μl/well) for 1 h and the absorbance was measured at 595 nm.

### Statistical analysis

The statistical analysis and graph drawing were performed in SPSS 20.0 for Windows. PFGE band patterns were generated with the Labworks 4.5 software package with 1% tolerance. Similarity coefficients were generated from a similarity matrix calculated with the Jaccard coefficient. Differences between the groups are assessed by two-tailed Mann-Whitney test. Correlations were analyzed by calculating Spearmann’s rho coefficient and presented as tables. The data is presented as bars showing mean values ± standard errors.

## Results

### Patients and bacterial strains

Sixty-eight patients were included in the study, 32 males and 36 females (male: female ratio 0.9:1). There were 27 CF patients treated as outpatients and inpatients. Their median age was 4.8 years (range 4 months to 34 years). Out of 41 non-CF patients, 27 (65.9%) were hospitalized in three intensive care units (pediatric medical, surgical and cardiothoracic), while the 14 others were treated on different specialized clinical wards. The median age of non-CF patients was 0.2 years (range 3 days to 15 years).

A total of 88 clinical isolates were examined. Forty-two isolates from CF patients were cultured from respiratory samples. Forty-six isolates from non-CF patients were cultured from a various sites (blood, bronchial washing fluid, endotracheal aspirates, sputum, urine, abdominal cavity drainage fluid, cough swabs, nose/throat secretions, wounds, etc. Single isolates were archived from each of 52 patients (16 CF and 36 non-CF) while from 16 additional patients (11 CF and 5 non-CF) more than one isolate were collected per patient.

### Genotyping of clinical isolates of *S*. *maltophilia*

**Fig 1:**
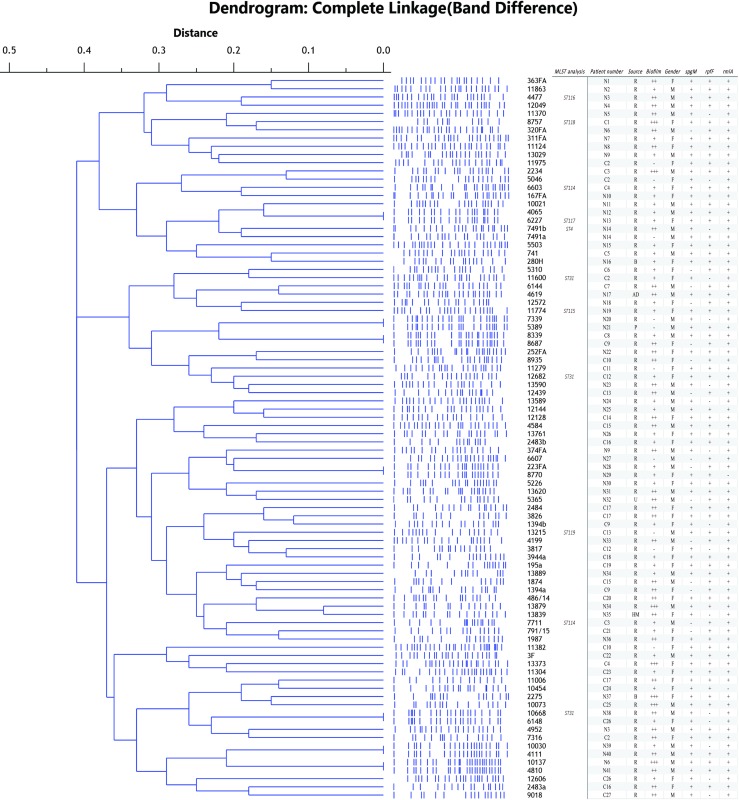
Phylogenetic analysis of obtained PFGE *Xba*I profiles of *S*. *maltophilia* clinical isolates. Distance showed above the dendrogram represents genetic relatedness between the analyzed strains. MLST analysis represents strains used for this analysis and obtained results. Patient number represents strains obtained from CF and non-CF patients, C and N, respectively. Isolates obtained from the same patients have identical patients number. Source represents site of isolation: R–respiratory tract, B–blood, HM–human milk. Strength of biofilm formed is presented with—–no biofilm, +–weak biofilm, ++–medium biofilm, +++–strong biofilm.

Sequencing of PCR products of amplified 16S rRNA gene confirmed all analyzed isolates as *S*. *maltophilia*, with identity ranging from 95% to 99% with *S*. *maltophilia* strains from the NCBI database. Genetic relatedness among the isolates was assessed by PFGE analysis, which revealed the existence of 11 pulsotypes. This result was obtained after analysis of PFGE profiles with SPSS program and construction of dendrograme (Fig 1). When the distance was ≤ 0.1 strains were considered genetically related. A subset of 11 isolates representing different pulsotypes was subject to MLST analysis; six of these proved to be novel (S1 Table). Three isolates (11600, 10668 and 12682), belong to ST31 as an isolate from Perth, Australia. One isolate (7491b), belongs to group ST4, comprising two other isolates from Europe. These previously identified STs were also clinical isolates of human origin. New allele sequences were deposited at the MLST Database hosted by the University of Freiburg, Germany, http://pubmlst.org/perl/bigsdb/bigsdb.pl?db=pubmlst_smaltophilia_isolates&page=query.

### Antibiotic susceptibility

Trimethoprim/sulfamethoxazole (TMP/SMX) demonstrated excellent inhibitory effect against all tested *S*. *maltophilia* isolates, which confirmed its potential clinical use. The MIC_50_ was ≤4 μg/ml, while the MIC_90_ was ≤32 μg/ml. For 60 strains (68.18%), the MIC_90_ was ≤10 μg/ml. All tested strains were sensitive to ciprofloxacin, chloramphenicol, tetracycline and levofloxacin (Table 1).

**Table 1:** Antibiotic susceptibility of the *S*. *maltophilia* clinical isolates (n = 88).

	Disc diffusion method			
Antimicrobial agents	Zone diameter interpretive criteria			Percentage of susceptible stains
	S	I	R	
Ciprofloxacin**	≥21	16–20	≤15	100%
Chloramphenicol*	≥18	13–17	≤12	100%
Tetracycline**	≥15	12–14	≤11	100%
Levofloxacin	≥17	14–16	≤13	100%
	Microdilution method			
	S	I	R	
TMP/SMX	≤2/38	-	≥4/76	100%

*—brakpoints for *E*. *coli* ATCC25922

**—breakpoints for *P*. *aeruginosa* ATCC27853

TMP/SMX—trimethoprim/sulfamethoxazole.

### Motility, biofilm formation and adhesion to mucin are in positive correlation

**Fig 2:**
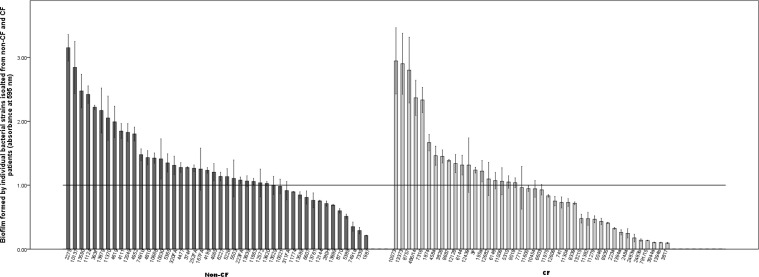
Biofilm formed by individual bacterial *Stenotrophomonas maltophilia* strains isolated from CF and non-CF patients.

Most of the analyzed strains were able to form biofilm with almost equal representation among CF and non-CF strains (Fig 2). The results of a biofilm formation assay on polystyrene showed that strong biofilm was formed by seven strains (7.95%), and only nine strains (10.2%) did not form biofilm. Moderate biofilm was formed by 37 strains (42.05%), while weak biofilm was formed by 35 strains (39.8%). Four out of seven strains forming strong biofilm were from CF patients, but among the 37 strains that formed moderate biofilm the slight majority were from non-CF patients. In general, no significant differences could be observed in biofilm formation concerning the source of strain isolation. Interestingly, this was not the case with strong biofilm producers, as all were respiratory isolates except one from a blood culture. PCR–based screenings of *rmlA*, *rpfF* and *spgM* genes revealed their presence in 86 strains (97.7%), 62 strains (70.4%) and 63 strains (71.6%), respectively. There was no difference regarding the presence of these biofilm-associated genes between CF and non-CF isolates. According to Spearmann’s rho coefficients (S2 Table), there was no statistical correlation between biofilm strength and the presence of *rmlA*, *rpfF* or *spgM* genes. However, the presence of both *rpfF* and *spgM* genes in the same strain was correlated with strong biofilm formation (p<0.05), while there was no correlation with other combinations of genes (*rmlA* + *rpfF* and *rmlA* + *spgM*). Furthermore, a negative correlation was observed between *rpfF* and *spgM* presence (p<0.05).

**Fig 3:**
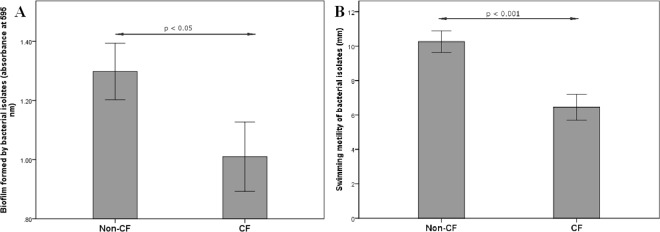
**Biofilm forming potential (A) and motility (B) of non-CF and CF *S*. *maltophilia* isolates.** Bars represent mean values ± standard errors.

**Fig 4:**
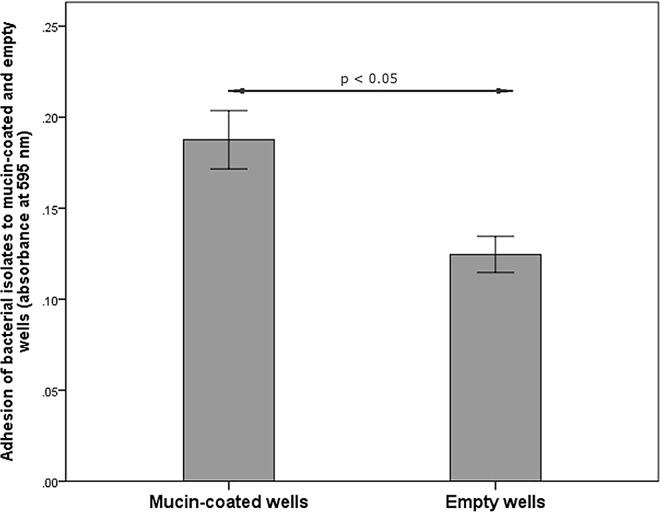
Adhesion of *S*. *maltophilia* isolates to mucin-coated and non-coated wells of microtiter plate. Bars represent mean values ± standard errors.

Out of 88 analyzed strains, only one demonstrated medium hydrophobicity of the cell surface (44% adherence to hexadecane) while in the other strains low hydrophobicity was confirmed. Swimming motility was observed in all tested strains while none of them showed swarming motility. According to Mann-Whitney test, non-CF isolates showed higher biofilm forming potential and motility than CF isolates (p = 0.021 and p = 0.0001, respectively) (Fig 3A and 3B). Mucin-adhesion ability was calculated as the ratio of absorbance at 595 nm, measured in mucin-coated wells against absorbance in control non-coated wells. There were no differences in mucin-adhesion ability between CF and non-CF isolates (graph not shown). Spearmann’s rho coefficients were calculated to check for correlations between the tested parameters. According to the Spearmann’s rho, motility has shown positive correlations (p < 0.01) with both biofilm formation and the mucin-adhesion ability of the strains (S3 Table). Besides comparing mucin-binding ability of CF and non-CF isolates, the ability of each individual strain to adhere to mucin was compared to its affinity to adhere to a plastic surface (mucin-coated vs. non-coated wells) (Fig 4). Mann-Whitney test revealed significantly higher adhesion of the isolates to mucin-coated compared to non-coated wells (p<0.05).

## Discussion

The prevalence of *S*. *maltophilia* has increased in hospitals worldwide simultaneously with the emergence of a myriad of other antibiotic resistant bacteria [1, 2]. In Serbia, decades of misuse of antibiotics due to poor and unenforced regulations, resulted in high prevalence of antibiotic resistant strains. Recently, Serbia was, along with other Balkan countries, indicated as a potential endemic region and the second common putative country of origin of isolates carrying the New Delhi Metallo-beta-lactamase-1 (NDM-1) gene, *bla*_NDM-1_ [22, 23]. Along with NDM-1 producers, inherently carbapenem-resistant organisms, including *S*. *maltophilia*, have been isolated in the hospital setting. Genomic variability among the 88 analyzed strains was high indicating the lack of clonal spread. This is also supported by the fact that 16 patients had more than one isolate and they were genetically different. Similar results were obtained in previous studies [24] but cases of hospital-associated infections have also been described [25]. MLST analysis identified two previously described STs, one from Europe and the other from Australia, and six novel STs, first described in this study (ST114, ST115, ST116, ST117, ST118 and ST119). However, the most dominant STs in Europe [16] were not detected among patients from Serbia. The determination of new STs is keeping with the high plasticity and capacity of bacteria to adapt to specific niches and develop new characteristics. Selective pressure imposed by conditions in the hospital environment could promote survival of certain STs with an adaptive advantage in this setting, which might lead to their clonal spread.

An increased number of reports on *S*. *maltophilia* resistant to TMP/SMX [26] have caused concerns, since TMP/SMX has been considered the main antibiotic for the treatment of *S*. *maltophilia*. What’s more, for *S*. *maltophilia* EUCAST set a breakpoint for TMP/SMX, even while the Clinical and Laboratory Standards Institute (CLSI) approved standards for levofloxacin, minocycline, ticarciline/calvulanate and ceftazidime. Then again, results obtained with TMP/SMX are the most reproducible, with no relation to the methods in susceptibility testing used [27]. In the Eastern and Southeast Europe, only data from Greece and Hungary are available [28, 29]. All of the *S*. *maltophilia* strains exhibited excellent susceptibility to TMP/SMX as well as chloramphenicol, ciprofloxacin, levofloxacin and tetracycline. This migh be, at least to some extent, atributed to the fact that these agants are generally not recommended for use in the children, hance the absence of selective preasure.

A positive correlation between motility, biofilm formation and adhesion to mucin was shown in our study. These results are different from those previously published [30], where authors did not find a correlation between motility and adhesion to non coated wells and biofilm formation. However, another study that had compared CF and non-CF clinical isolates suggested that motility was crucial for biofilm development in CF isolates [19]. Factors affecting adhesion of *S*. *maltophilia* to mucin and clinical relevance of this activity have not been elucidate yet. According to our results, clinical isolates of *S*. *maltophilia* exhibited the ability to adhere to mucin. So far only in one study it was shown that *S*. *maltophilia* could adhere to mouse tracheal mucus with the help of flagella [31].

Biofilm formation in bacteria is a multifactorial event that depends on surface characteristics, motility of strains, genes involved in biofilm formation, and other factors, and can be correlated with a higher level of resistance to antibiotics [32]. Different factors influenc biofilm formation in *S*. *maltophilia*, namely SmeYZ efflux pump that also confers resistance to antimicrobials [33], iron level in the media [34], and histidin kinase and BfmAK system [35]. Apart from biofilm formation, other physiological functions (such as swimming moility, oxidative stres regulation, etc.) are regulated through aforementioned mechanisms. Interestingly, our study revealed a positive correlation between the simultaneous presence of genes *spgM* and *rpfF* in a strain and strong biofilm production (4 x ODc ˂ OD). This correlation was not affected by the presence or absence of the *rmlA* gene. The negative correlation observed between *spgM* and *rpfF* genes could indicated that the presence of either one of these genes is required for biofilm formation, but the presence of both could lead to stronger biofilm production. However, further examination at the level of *spgM* and *rpfF* expression is required to support this assumption. An interesting observation of this study is the higher motility and biofilm-forming potential of non-CF versus CF isolates. Although the loss of motility of CF pathogens has already been described as part of their adaptation process to the CF environment, the decrease in biofilm formation is not easily explained. *Pseudomonas aeruginosa* the major pathogen in the CF lung due to biofilm-growing mucoid (alginate-producing) strains, which confer resistance to the host defense mechanism. Pompilio et al. [36] reported the prevalence of *P*. *aeruginosa* in mixed biofilm communities formed by *P*. *aeruginosa* and *S*. *maltophilia* in the CF lungs. Actually, *S*. *maltophilia* stimulates biofilm formation by *P*. *aeruginosa*. This altruistic behavior of *S*. *maltophilia* facilitates its survival in mixed biofilms. Considering this, it could argued that *S*. *maltophila* strains which are poor biofilm producers more sucessfully survive in the CF lungs. Otherwise, they would be outcompeted by more aggressive *P*. *aeruginosa* strains. This might explain the higher incidence of poor biofilm-producing strains among CF *S*. *maltophilia* isolates in our study.

In summary, this work represents the first epidemiological study of clonal relatedness and antibiotic resistance of *S*. *maltophilia* clinical isolates in Serbia. Clonal diversity detected in this study indicates low cross-transmission of *S*. *maltophilisa* in the hospital settings. The susceptibility testing gained unremarkable results, as strains were universally susceptible to the tested antibiotics. A comparison of phenotypic characteristics of CF and non-CF isolates suggested that there was a difference between the two populations. Six novel *S*. *maltophilia* STs were revealed while none of the STs prevalent in Europe were identified. This study accentuates the need for continuous surveillance for *S*. *maltophilia* in hospital settings in Serbia and monitoring their evolution towards antibiotic resistance.

## Supporting Information

S1 Table Sequence type (ST) of 11 *Stenotrophomonas maltophilia* clinical isolates. *—C–CF patients, N–non-CF patients.
(DOC)

S2 Table Correlations between presence of PCR signals for *spgM*, *rpfF* and *rplA* genes and biofilm formation in *S*. *maltophilia* isolates according to Spearmann’s rho coefficients. *—correlation is significant at the 0.05 level, (2-tailed), **—correlation is significant at the 0.01 level, (2-tailed).
(DOC)

S3 Table Correlations between tested physiological parameters of *S*. *maltophilia* isolates according to Spearmann’s rho coefficients. *—correlation is significant at the 0.05 level, (2-tailed), **—correlation is significant at the 0.01 level, (2-tailed).
(DOC)
