# Supplementary material for: Genotypic and Phenotypic Characterization of Stenotrophomonas maltophilia Strains from a Pediatric Tertiary Care Hospital in Serbia
Source: PLoS One. 2016 Oct 31;11(10):e0165660. doi: 10.1371/journal.pone.0165660 (PMC5087882; doi:10.1371/journal.pone.0165660)
Supplement: S1 Table — *—C–CF patients, N–non-CF patients. (DOC) [file pone.0165660.s001.doc]

**S1 Table. Sequence type (ST) of 11 *Stenotrophomonas maltophilia*** clinical isolates.

| Strain | Patient number* | *atpD* | *gapA* | *guaA* | *mutM* | *nuoD* | *ppsA* | *recA* | ST |
| --- | --- | --- | --- | --- | --- | --- | --- | --- | --- |
| 11600 | C2 | allele 3 | allele 4 | allele 24 | allele 7 | allele 7 | allele 22 | allele 7 | 31 |
| 7491b | N14 | allele 1 | allele 4 | allele 7 | allele 7 | allele 28 | allele 19 | allele 6 | 4 |
| 10668 | N38 | allele 3 | allele 4 | allele 24 | allele 7 | allele 7 | allele 22 | allele 7 | 31 |
| 12682 | C12 | allele 3 | allele 4 | allele 24 | allele 7 | allele 7 | allele 22 | allele 7 | 31 |
| 6603 | C4 | allele 3 | allele 4 | allele 18 | allele 1 | allele 7 | allele 20 | allele 1 | 114 |
| 7711 | C3 | allele 3 | allele 4 | allele 18 | allele 1 | allele 7 | allele 20 | allele 1 | 114 |
| 11774 | N19 | allele 3 | allele 1 | allele 84 | allele 57 | allele 25 | allele 82 | allele 6 | 115 |
| 4477 | N3 | allele 3 | allele 1 | allele 84 | allele 58 | allele 25 | allele 82 | allele 6 | 116 |
| 6227 | N13 | allele 1 | allele 4 | allele 43 | allele 3 | allele 70 | allele 83 | allele 7 | 117 |
| 8757 | C1 | allele 2 | allele 2 | allele 93 | allele 59 | allele 63 | allele 69 | allele 5 | 118 |
| 13215 | C13 | allele 4 | allele 76 | allele 92 | allele 5 | allele 70 | allele 84 | allele 9 | 119 |

* - C – CF patients, N – non-CF patients
