# Supplementary material for: Genotypic and Phenotypic Characterization of Stenotrophomonas maltophilia Strains from a Pediatric Tertiary Care Hospital in Serbia
Source: PLoS One. 2016 Oct 31;11(10):e0165660. doi: 10.1371/journal.pone.0165660 (PMC5087882; doi:10.1371/journal.pone.0165660)
Supplement: S2 Table — *—correlation is significant at the 0.05 level, (2-tailed), **—correlation is significant at the 0.01 level, (2-tailed). (DOC) [file pone.0165660.s002.doc]

**S2 Table. Correlations between presence of PCR signals for *spgM*, *rpfF* and *rplA* genes and biofilm formation in *S. maltophilia* isolates according to Spearmann’s rho coefficients.**
